# MV140 mucosal bacterial vaccine improves uropathogenic *E. coli* clearance in an experimental model of urinary tract infection

**DOI:** 10.21203/rs.3.rs-2992611/v1

**Published:** 2023-06-07

**Authors:** Paula Saz-Leal, Marianne M. Ligon, Carmen M. Diez-Rivero, Diego García-Ayuso, Soumitra Mohanty, Laura Conejero, Annelie Brauner, José L. Subiza, Indira U. Mysorekar

**Affiliations:** 1 Inmunotek, S.L. Alcalá de Henares, Madrid, Spain.; 2 Department of Obstetrics and Gynecology, Washington University School of Medicine, St. Louis, MO, USA.; 3 Department of Microbiology, Tumor and Cell Biology, Karolinska Institutet, Stockholm, Sweden.; 4 Division of Clinical Microbiology, Karolinska University Hospital, Stockholm, Sweden.; 5 Department of Medicine, Section of Infectious Diseases, Baylor College of Medicine, Houston, TX.; 6 Department of Molecular Virology and Microbiology, Baylor College of Medicine

## Abstract

MV140 is a mucosal vaccine of inactivated whole bacteria (*E. coli, K. pneumoniae, E. faecalis, P. vulgaris*) with clinical efficacy against recurrent urinary tract infections (UTIs). Here, MV140 was evaluated in a murine model of acute uropathogenic *E. coli* (UPEC)-induced UTI using the UTI89 strain. MV140 vaccination resulted in UPEC clearance, concomitant with increased influx of myeloid cells in urine, CD4+ T cells in the bladder, and a systemic adaptive immune response to both MV140-containing *E. coli* and UTI89.

## INTRODUCTION

Urinary tract infections (UTIs) are common and recur frequently. They are primarily of bacterial origin (uropathogenic *Escherichia coli -*UPEC- being the most common causative agent), mostly affect women and acute infections are commonly treated with antibiotics (1, 2). UTIs can become recurrent and may require long-term antibiotic prophylaxis, which produces numerous side effects and contributes to the emergence of resistant strains; the latter in turn favors further recurrences (1–3). Thus, there continues to be an urgent need to find non-antibiotic therapies to limit these frequent and recalcitrant infections.

MV140 is a sublingual vaccine composed of selected strains of inactivated whole-cell bacterial species (V121 *E. coli*, V113 *K. pneumoniae*, V125 *E. faecalis* and V127 *P. vulgaris*) that has shown long-term efficacy (up to a year of follow-up) in the prevention of recurrent UTI in women (4). Preclinical studies showed that MV140 induces humoral and systemic CD4_+_ T cell responses against MV140-contained bacteria following sublingual or intranasal vaccination (5, 6). However, there is no direct evidence that MV140 can protect against UTIs in experimental models, nor that the vaccine response can be protective against UPEC strains not contained in the MV140 formulation. In this study, we used an experimental murine model of UPEC-induced UTI with a clinical *E. coli* isolate, UTI89, to evaluate the protection and the local and systemic immune responses elicited by MV140.

## RESULTS

**Figure 1.**
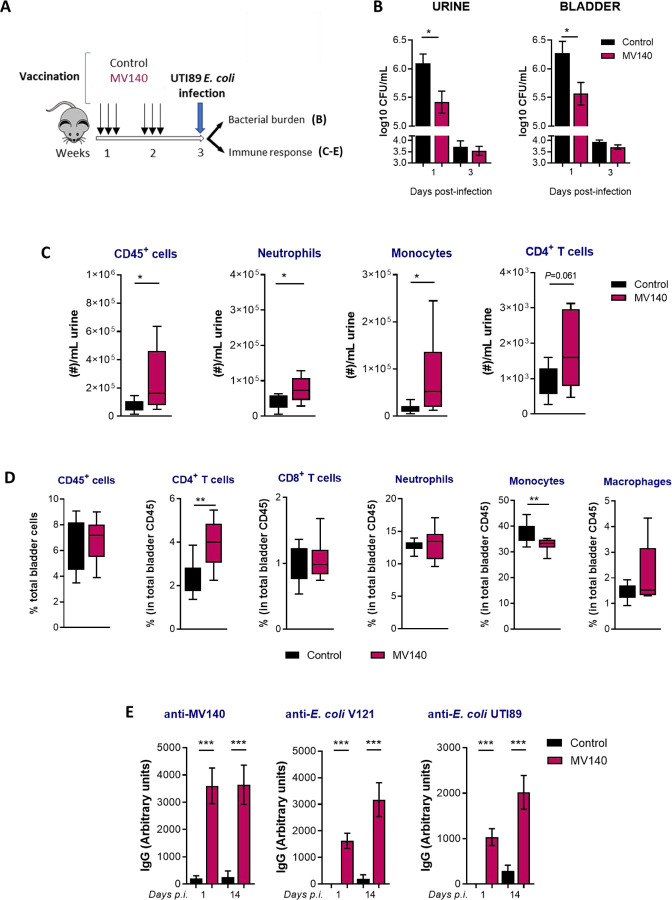
MV140 mucosal vaccine confers early protection against uropathogenic *E. coli* UTI89 infection, accompanied with heightened humoral and local cellular immunity. **(A)** Scheme of the intranasal immunization protocol and subsequent transurethral infection with UTI89 uropathogenic *E. coli*. **(B)** Bacterial load in the urine (left panel) and bladder (right panel) at indicated timepoints upon infection. Mean ± SEM of three (bladder) or eight (urine) independent experiments is shown (n≥8). **(C-D)** Cellular response including CD45+ cells, neutrophils, monocytes, macrophages and T lymphocytes in urines (C) and bladders (D) at 24 hours post-infection, analyzed by flow cytometry. Data are shown as boxplots and min-max whiskers of two independent experiments (n≥8). **(E)** Specific IgG antibodies against MV140, MV140-containing *E. coli* V121 and uropathogenic *E. coli* UTI89 generated in mice at indicated timepoints post-infection. Data are shown as arbitrary units, calculated as described in Methods. Mean ± SEM of 2–3 independent experiments per timepoint (n≥10). **(B-E)** Mice were immunized with control (vaccines excipients, black) or MV140 (magenta) and subsequently infected as stated in A. * *P*<0.05, ** *P*<0.01, *** P<0.001, unpaired Student’s t-test or Mann-Whitney test, according to normal distribution assessed by Shapiro-Wilk test. p.i., post-infection. CFU, colony forming units.

Transurethral immunization of mice with attenuated UPEC strains confers nonspecific protection (7) and intranasal immunization with different UPEC antigens has been shown to induce high levels of immunoglobulins in urine (8). To test whether intranasal (i.n.) immunization with MV140 would be an effective means of eliciting a mucosal immune response in the bladder and UTI protection, mice were tested with MV140 or vaccine excipients (control) and subsequently challenged with UTI89 (Figure 1A) following a well-established murine model of acute UTI (cystitis) described in Hung et al. (9). To evaluate the course of infection, bacterial load was determined during the acute phase (1–3 days post-infection, p.i.). Bacterial load was significantly lower in both urine and bladder of MV140-vaccinated mice than in control mice at 24h p.i. (Figure 1B). As it may be expected, no differences were shown on day 3 post infection, when MV140 and control mice had already cleared the infection in this model.

A local immune cell response with a higher influx of CD45_+_ cells, mainly neutrophils and monocytes, was seen 24h p.i. in urines of MV140-vaccinated mice compared to controls (Figure 1C). At this timepoint, while total numbers of urine CD4^+^ T cells were close to a significant increase in MV140-vaccinated mice (Figure 1C), a clear rise of bladder CD4_+_ T cells relative to the CD45+ compartment was observed (Figure 1D).

Given the rapid bacterial clearance kinetics in MV140-immunized mice compared to controls, we next assessed the differential production of antimicrobial peptides by the urothelium. Cramp (cathelicidin-related antimicrobial peptide) and psoriasin (S100a7a) levels which have been shown to be activated during a UTI (10, 11) were analyzed. A slight close-to-significant increase in bladder expression of psoriasin (p = 0.051) was detected by qPCR at 24h p.i. in MV140-immunized mice; however, no change was noted at the protein level (Supplementary Figure 1).

On the other hand, MV140-vaccinated and infected mice had significantly higher levels of serum IgG antibodies, against both UTI89 and V121 (included in MV140) *E. coli* strains, as well as against the whole MV140 bacterial mixture, compared to unvaccinated but infected control mice. This antibody response was already present early upon infection (24h p.i.) and slightly increased two weeks later (day 14 p.i.) (Figure 1E).

**Figure 2.**
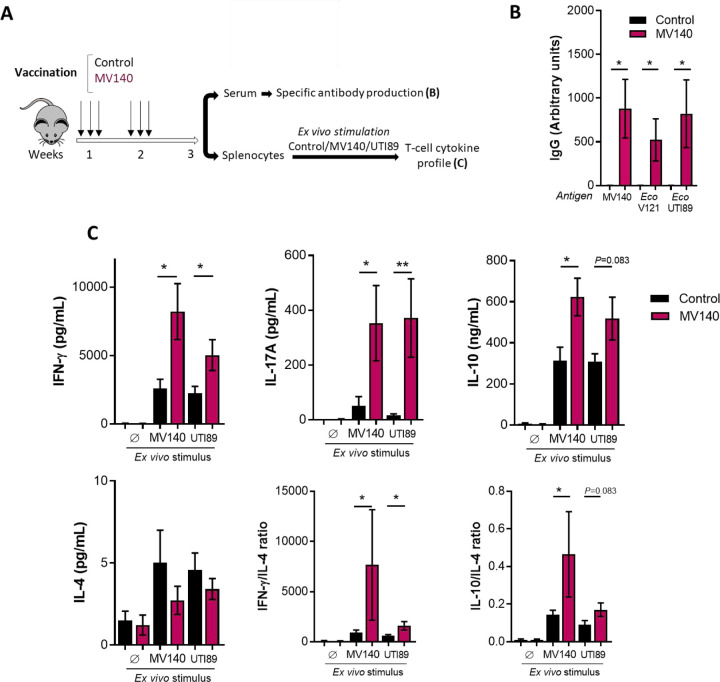
Mucosal vaccination with MV140 induces the generation of systemic adaptive immune responses. **(A)** Scheme of the intranasal immunization protocol with control (vaccine excipients, black) or MV140 (magenta) and analysis of induced systemic immune response. **(B)** Serum specific IgG antibodies against MV140, MV140-containing *E. coli (Eco)* V121 and uropathogenic *E. coli (Eco)* UTI89 generated in mice according to A. Data are shown as arbitrary units, calculated as described in Methods. Mean ± SEM of 3 independent experiments is shown (n=10). **(C)** Cytokine production (IFN-γ, IL-4, IL-17A and IL-10) in supernatants of splenocytes isolated from mice immunized and restimulated according to A. Mean ± SEM of two independent experiments (n=7) is shown. **(B,C)** * *P*<0.05, ** P<0.01, *** P<0.001, unpaired Student’s t-test or Mann-Whitney test comparing between treatmen groups, according to normal distribution assessed using Shapiro-Wilk test. OD, optical density (arbitrary units); Ø, unstimulated.

Having demonstrated the protective effect of MV140 in this experimental model, we next wanted to address the specific response to UTI89 prior to infection in MV140-immunized mice (Figure 2A). Serum IgG antibodies and T helper (Th) 1 (IFN-γ), Th2 (IL-4), Th17 (IL-17) and regulatory T cell (IL-10) responses were assessed. Serum levels of IgG antibodies against MV140, V121 and UTI89 were similarly induced in MV140-immunized mice (Figure 2B). Likewise, splenocytes from mice immunized with MV140 produced significantly higher amounts of IFN-γ, IL-17 and IL-10 i*n vitro* than control mice following stimulation with either MV140 or heat-killed UTI89 at comparable rates (Figure 2C). Notably, IL-17 response was strongly induced by both stimuli compared to non-immunized mice. By contrast, this was not the case for IL-4, where a trend towards lower production in response to both MV140 and UTI89 was observed (Figure 2C). This resulted in the IFN-γ/IL-4 ratio being significantly higher after stimulation with MV140 or UTI89 (Figure 2C).

## DISCUSSION

MV140 is a mucosal vaccine that has been shown to prevent UTI recurrences in different clinical settings (4, 12–14) including a randomized placebo-controlled clinical trial (4, 12–14). Preclinical studies, both *in vitro* and *in vivo*, indicate that MV140 is a potent activator of DCs, endorsing these cells the ability to induce Th1, Th17 and regulatory T (Treg) cells (5, 6, 15). However, direct evidence of MV140 conferring protection in an experimental UTI infection model was lacking. Here we describe in a well-established model of UTI with the UPEC strain UTI89 that mice immunized with MV140 show an early reduction of bacterial load in urine and bladder compared to non-immunized controls. This was accompanied by an increased local influx of myeloid and CD4_+_ T cells in MV140-immunized mice, pointing to an adaptive immune cell response. The rapid T cell response after infection clearly points to a prior induction due to vaccination with MV140. This is supported by previous works (5, 6, 15) and the present study, which show the induction of a systemic T-cell response in mice immunized with MV140. Notably, the extent of Th1/Th17 responses were very similar against *E. coli* strains V121 or UTI89, the latter not included in the formulation of MV140. This could be also seen at the humoral level, as serum levels of IgG antibodies against V121 or UTI89 strains were similarly increased in MV140-immunized mice.

Although the induced antibody response might be involved in MV140 protection, the key role of T cells, particularly Th17, in mucosal infections due to extracellular bacteria has been highlighted elsewhere (16). Activated Th17 cells control the influx of neutrophils into mucosal tissues by releasing inflammatory cytokines such as TNF-α and IL-17, which in addition may induce production of neutrophil-attracting chemokines by epithelial cells (17) and antimicrobial peptides (18). In this regard, bladder cells from MV140-immunized mice sublingually produce TNF-α in response to MV140 (5). The present results may also point to an increase in psoriasin at the mRNA level in the bladder after MV140 immunization but, without detectable increase in tissue protein expression in this model.

Of particular interest is the down-regulation of the Th2 immunity in mice immunized with MV140 after stimulation with V121 or UTI89, given the negative role attributed to this response in recurrent bladder infections (19). Such down-regulation was previously noted when analyzing *in vitro* the T cell-polarizing properties of human monocyte-derived DCs primed with MV140 (5, 6). Th1 immunity induced in MV140-immunized mice, together with the IL-10 response (5, 6, 15), may also be involved in down-regulating established Th2 responses *in vivo* (16).

In general, T cell responses are serotype independent (20) and shared/cross-reactive T cell epitopes are frequent even between different enterobacteria causing UTIs (21), which supports the effectiveness of MV140 in the clinical setting against infections of diverse bacterial origin (22). As shown herein, MV140 responses are triggered against V121-containing *E. coli* strain, but also against specific clinically infectious uropathogenic isolates such as UPEC UTI89. Altogether, considering the results in terms of protection, T-cell and IgG responses, it can be concluded that MV140 is protecting against UPEC in a non-strain specific manner.

The mucosal route was chosen for treatment delivery of MV140 because it is described to induce both systemic and mucosal immunity (including the genitourinary tract) (5, 6, 15, 23). This is highly desirable to provide an efficient protection against pathogen invasion through mucosal tissues as shown for other whole-cell bacterial formulations (24–26). Recently, a sublingual vaccine with peptides derived from the UPEC strain CFT073 has been shown to be effective against an experimental challenge with this particular strain, although the efficacy of this vaccine against other UPEC strains remains to be evaluated (27).

To summarize, our data indicate that MV140 induces strong systemic and local adaptive immunity in the bladder against a clinical UPEC isolate, which correlates with early bacterial clearance after infection.

## METHODS

### Mice and in vivo models

Female C57BL/6 Wild-type mice (8- to 10-week-old) from Jackson laboratory were used. Mice were maintained under specified pathogen-free conditions in a barrier facility under a 12 h light-dark cycle. All experimental procedures were approved by the animal studies committee of Washington University in St. Louis School of Medicine (Animal Welfare Assurance #A-3381–01).

As immunization protocol, mice were intranasally (i.n.) administered with 50 μL of MV140, a suspension of equal amounts of whole-cell heat-inactivated bacteria: V121 *Escherichia coli*, V113 *Klebsiella pneumoniae*, V125 *Enterococcus faecalis*, V127 *Proteus vulgaris* (UROMUNE^®^, Inmunotek S.L., Spain) at 300 Formazin Turbidity Units (FTU, ~10^9^ bacteria/mL), or vaccine excipients (control) diluted in phosphate-buffered saline (PBS; Sigma-Aldrich), 3 times a week for 2 weeks, and rested for an additional week.

For experimental UTI, UTI89, a clinical UPEC isolate from a patient with recurrent cystitis (28) was grown statically for 17h in Luria-Bertani broth (LB, Tryptone 10 g/L, Yeast extract. 5 g/L. and NaCl 10g/L, Sigma-Aldrich) at 37°C prior to infection. Mice were anesthetized and inoculated via transurethral catheterization with 10^7^ colony forming units (CFUs) of UTI89 in PBS, as published elsewhere (9). Urines and bladders were collected at indicated timepoints (24–72 hours post-infections, h.p.i) and spotted onto LB-agar plates to measure bacterial titers.

### Splenocyte ex vivo stimulation assay

Spleens were used to prepare single cell suspensions following conventional protocols. Cells (1.5·10^6^/mL) were plated in 96-well plates (200 μL final volume; Corning) and in vitro stimulated for 72h with heat-killed UTI89, MV140 (both 3 FTU, ~10^7^ bacteria/mL) or control, and cytokine production (IL-17A, IFN-γ, IL-4, and IL-10) measured in cell-free culture supernatants by Multiplex cytokine assay (Bio-plex, Bio-Rad Laboratories), following manufacturer’s instructions.

### Serum antibody production

Specific IgG for MV140, MV140-containing strain *E. coli*, or UTI89 were determined by Enzyme Linked Immunosorbent Assay (ELISA) from serum samples obtained at indicated timepoints. Briefly, 96-well non-tissue culture-treated plates (Greiner Bio-One) were pretreated with poly-L-lysine (Sigma-Aldrich) for 1h under UV light. Then, plates were coated with the heat-inactivated whole cell bacteria mixture MV140, or heat-killed V121 *E. coli* or UTI89 (all at 450 FTU) overnight at 4°C, and, subsequently, incubated with mouse serum for 2h at room temperature (RT). Specific Igs were detected using biotin rat anti-mouse IgG, peroxidase and o-phenylenediamine dihydrochloride (all from Sigma-Aldrich). Plates were read at 492 nm (Synergy Mx, Biotek).

### Flow cytometry

Bladders were digested at 37°C for 30 minutes in RPMI-1640 with 10mM HEPES, collagenase D, and DNAse (all Sigma-Aldrich), forced through a 70 μm cell strainer (Corning), and washed with 5% fetal bovine serum (FBS, ThermoFisher Scientific) in PBS. Urines were diluted in fluorescence-activated single cell sorting (FACS) Buffer (PBS supplemented with 5 mM EDTA and 3% FBS) for 30 minutes (pH neutralization), prior to staining. Single cell suspensions resuspended on ice-cold FACS Buffer were stained with anti-CD45 eFluor450 (eBioscience), anti-CD3-A700 (eBioscience), anti-CD4-PE/Cy7 (BioLegend), anti-CD8-BrilliantViolet605 (BioLegend), anti-CD11b-PE/Cy5 (eBioscience), anti-Ly6C-APC/Cy7 (BioLegend), anti-Ly6G-FITC (BioLegend), anti-F4/80-PE (eBioscience), anti-CD64-APC (BioLegend) and 7-AAD (BioLegend). Purified anti-FcɣRIII/II (BioLegend) was used to block Fc-receptors. Data was acquired on LSR II flow cytometer (BD) and analyzed with FlowJo software v10.0 (BD).

### Antimicrobial peptide production

For immunofluorescence analysis, bladders were fixed in methacarn (60% methanol, 30% chloroform, 10% acetic acid) and embedded in paraffin. Bladder sections were deparaffinized using neoclear and rehydrated, pretreated with 0.3% Triton X-100 in PBS (PBS-T), and boiled in citrate buffer, 1 mM EDTA, 10 mM TRIS, 0.05% Tween 20 (pH 9). Sections were rinsed with PBS-T, treated with FX Signal Enhancer (Invitrogen) at RT for 30 min and blocked for 60 mins with sera. Further, anti–psoriasin (1:200; Santacruz) and anti-UPIIIa (1:200; Santa Cruz) were incubated overnight at 4^°^C. Sections were washed with PBS-T and further incubated with respective secondary Alexa Fluor– conjugated antibody (Invitrogen) in 1:600 for 1.5h at RT, followed by staining with DAPI for 15 min, washed and mounted in Fluoromount G (Southern Biotech). Slides were analyzed with a Zeiss 700 confocal microscope and fluorescence intensity quantified with the ImageJ software.

For RT-qPCR, bladders were flash frozen, and RNA extracted using TRIzol reagent (Invitrogen) according to manufacturer protocol followed by gDNA digestion with TURBO DNA-free kit (Invitrogen). cDNA was generated using Superscript II Reverse Transcriptase (Invitrogen). qPCR was performed with standard SYBR green (Applied Byosistems) on a Rotor-Gene PCR cycle (Corbett Life Science). Gene specific primers Cramp (forward: AATTTTCTTGAACCGAAAGGGC, reverse: TGTTTTCTCTCAGATCCTTGGGAGC), S100a7a (forward: GCTCGTTTAGTGAACCGTCAG, reverse: GGAGTCCTCCACTGGTGTGT) and Actb (forward: CTGTCCCTGTATGCCTCTG, reverse: ATGTCACGCACGATTTCC) were used. Fold-changes were calculated using Ct method and normalized internally to respective control.

### Statistical analysis

The statistical analysis was performed using Prism (GraphPad Software). Statistical significance for comparison between treatment groups was determined using Mann-Whitney or unpaired Student’s t tests, according to normal distribution evaluated by Shapiro-Wilk test. Outliers were identified by means of Tukey’s range test. For antibody analysis, arbitrary units were calculated from the optical density (OD) values according to standard curves, after subtracting the OD values of blank and adjusting by the serum dilutions performed. Differences were considered significant at p < 0.05. Except when specified, only significant differences are shown.

## Supplementary Material

1

## Data Availability

All data generated during this study are available from the corresponding author upon request.
