# Supplementary material for: MV140 mucosal bacterial vaccine improves uropathogenic E. coli clearance in an experimental model of urinary tract infection
Source: Res Sq. 2023 Jun 7:rs.3.rs-2992611. Preprint. [Version 1] doi: 10.21203/rs.3.rs-2992611/v1 (PMC10275044; doi:10.21203/rs.3.rs-2992611/v1)
Supplement: 1 [file NIHPPRS2992611V1-supplement-1.pdf]

Supplementary Figures

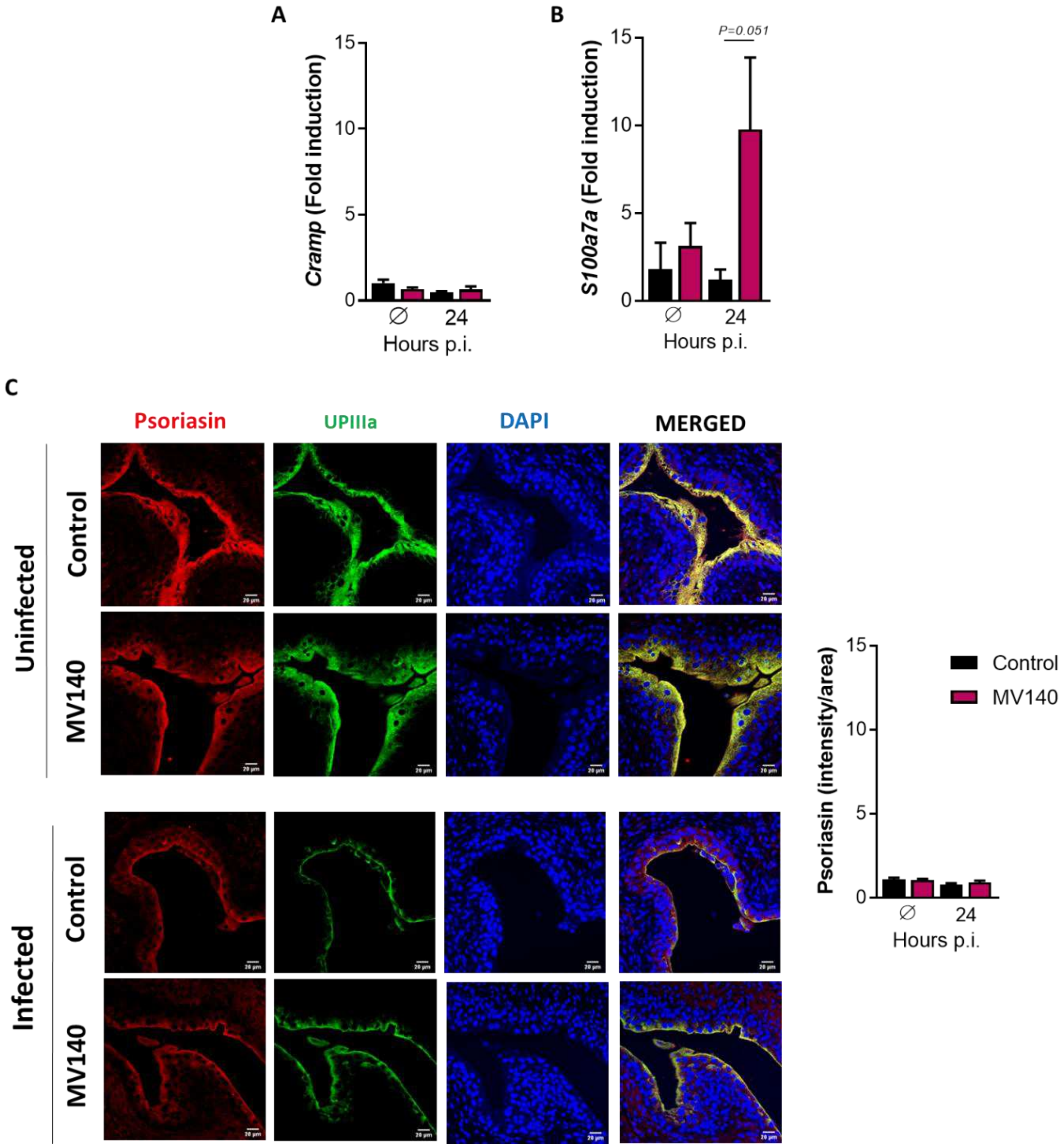

Supplementary Figure 1. Mucosal bacterial vaccination does not significantly modulate antimicrobial peptide production in the bladder urothelium following infection. (A,B) Antimicrobial peptide relative expression (*CRAMP, S100A7A* – Human psoriasin-coding gene) in total bladder at 24 hours post-infection, analyzed by q-PCR. Fold induction vs. control uninfected mice is represented. Mean + SEM of one (uninfected) or two (24 h p.i.) independent experiments is shown (n≥2). (C) Psoriasin protein expression in bladder urothelium at 24 hours post-infection, analyzed by immunofluorescence. Quantification of intensity per area from 3-5 random view fields is represented. Mean ± SEM of one (uninfected) or two (24 h p.i.) independent experiments is shown (n≥3). (A-C) Mice were immunized with control (vaccine excipients,

black) or MV140 (magenta) and subsequently infected as stated in Figure 1A. Normal distribution was assessed using Shapiro-Wilk test. *P* values were calculated by unpaired Student's t-test comparing between treatment groups were found. Ø, uninfected; p.i., post-infection; UPIIIa, uroplakin IIIa.
